# Sustained-Release Microneedles for Local Delivery of Antibacterial Peptide in Acne Therapy

**DOI:** 10.3390/polym18101250

**Published:** 2026-05-21

**Authors:** Jingyu Gao, Zhangyong Si, Mengdi Xu, Shengyu Zhang, Fan Fan, Feng Zhou, Jiantao Zhang

**Affiliations:** 1 Laboratory of Advanced Theranostic Materials and Technology, Ningbo Institute of Materials Technology and Engineering, Chinese Academy of Sciences, Ningbo 315300, China; gaojingyu@nimte.ac.cn (J.G.);; 2 Ningbo Cixi Institute of Biomedical Engineering, Ningbo 315300, China; 3 Zhejiang Key Laboratory of Biopharmaceutical Contact Materials, Ningbo 315300, China

**Keywords:** microneedles, acne, sustained-release, transdermal delivery, antibacterial peptide

## Abstract

Acne is a prevalent chronic inflammatory skin disorder with a high recurrence rate, in which *Propionibacterium acnes* (*P. acnes*) plays a key pathogenic role by colonizing subepidermal pilosebaceous units. The stratum corneum limits drug penetration, rendering conventional topical therapies ineffective. Herein, we report a detachable sustained-release microneedle system named Bacitracin@Hyaluronic Acid–Zein Microneedle (Bac@HA-ZMN) for localized antibacterial delivery in acne therapy. This microneedle patch consists of a dissolvable HA base and zein-based indwelling microneedle tips loaded with bacitracin (Bac) against *P. acnes*. Mechanical testing showed an average fracture force of 1.6 N per needle tip (*n* = 100), sufficient for skin insertion. The needle tips enabled Bac delivery to a depth of approximately 500 μm. In vitro transdermal studies demonstrated a cumulative release of 76.1% within 96 h, significantly higher than that of the control group (14.2%). In a murine acne model, the Bac@HA-ZMN treatment group showed a significantly smaller lesion area than the control group, and the immunohistochemical positive expression areas of the inflammatory factors IL-8, MMP-2, and TNF-α were reduced to 0.79%, 4.12%, and 2.14%, respectively, which was caused by the inhibitory effect of Bac on *P. acnes*. These results demonstrated Bac@HA-ZMN as a promising localized, sustained antibacterial delivery platform for acne treatment.

## 1. Introduction

Acne is one of the most common inflammatory skin disorders worldwide and affects individuals across a wide age range, particularly adolescents [1]. A key pathogenic contributor is *Propionibacterium acnes* (*P. acnes*), which predominantly resides in subepidermal pilosebaceous units, making eradication by topical therapy challenging [2,3]. The stratum corneum imposes a formidable barrier to drug transport; therefore, conventional topical agents (e.g., antibiotics, retinoids, benzoyl peroxide, azelaic acid, and salicylic acid) often exhibit limited penetration into lesion sites and require frequent or prolonged application, which may cause irritation and compromise adherence [4,5,6]. Photodynamic therapy (PDT) has emerged as an effective physical modality for the treatment of acne; however, it is commonly associated with considerable procedural pain and phototoxic adverse effects, such as burning sensation, erythema, and edema [7]. Hormonal therapy, including combined oral contraceptives (COCs) and anti-androgen agents, also plays an important role in acne management, with anti-androgen agents reducing acne by inhibiting sebum production and COCs being particularly beneficial for women with contraceptive needs, menorrhagia, or an inadequate response to standard therapies [4]. Therefore, given the biological characteristics of *P. acnes* and the growing concern regarding the emergence of antibiotic-resistant bacteria resulting from antibiotic misuse, there is a pressing need to develop an ideal therapeutic strategy for acne. Such a strategy should be capable of bypassing the skin barrier and providing sustained, localized antibacterial activity at the subepidermal lesion site, while minimizing systemic exposure and improving patient compliance [2]. While electrospun nanofiber-based formulations have been investigated for acne treatment, their effectiveness is often hindered by limited skin penetration and inadequate localization within the pilosebaceous unit, highlighting the need for more efficient transdermal delivery strategies such as microneedles [8,9]. In this context, microneedle patch-mediated drug delivery represents a promising approach to fulfilling these requirements.

Microneedle-based transdermal delivery systems can physically breach the stratum corneum in a minimally invasive manner [10,11], enabling direct drug deposition into targeted skin layers [12,13,14]. In particular, designs incorporating a dissolvable base with indwelling needle tips allow the tips to remain embedded after patch removal, thereby forming local depots for sustained release and prolonged therapeutic action at the lesion site [15,16,17]. Material selection is crucial for achieving both mechanical integrity and controlled release [18,19,20]. Zein, a hydrophobic plant-derived protein, provides robust mechanical strength and can regulate drug diffusion to enable sustained release [21,22,23]. Furthermore, studies have confirmed that it possesses certain antibacterial activity [24,25,26,27]. Hyaluronic acid (HA), a highly hydrophilic and biocompatible polysaccharide, is suitable for a rapidly dissolvable base that facilitates detachment after insertion [28,29]. The aforementioned functional materials were separately incorporated into the tip and base of the microneedles to establish a functionally compartmentalized architecture. The needle tips were designed to possess sufficient mechanical strength for effective skin penetration and stable in situ retention, whereas the base served as a supporting structure and was capable of controlled detachment following insertion, thereby facilitating the sustained delivery of the antimicrobial peptide in a coordinated manner.

With the increasing concern over resistance to topical antibiotics in acne treatment, there is a growing need to explore novel local antibacterial strategies with mechanisms distinct from those of conventional agents [30,31]. Although bacitracin is not a guideline-recommended first-line anti-acne therapy, it exhibits activity against Gram-positive bacteria and acts by interfering with cell wall precursor cycling, rather than inhibiting bacterial protein synthesis as clindamycin does [32,33]. This mechanistic difference suggests a lower likelihood of direct overlap with common clindamycin resistance pathways [34,35]. Therefore, bacitracin may serve as a promising investigational topical antibacterial candidate in the context of rising antibiotic resistance [36]. It should be noted, however, that bacitracin is not intended to replace established anti-acne agents such as benzoyl peroxide, which remains a cornerstone therapy due to its strong antibacterial efficacy and low propensity to induce classical antibiotic resistance. Although microneedle-based delivery systems have been widely investigated for the transdermal or intradermal administration of antimicrobial agents and therapeutic peptides [37,38], many previously reported platforms primarily rely on rapid dissolution after skin insertion, resulting in relatively short drug retention and limited capacity for sustained local treatment [38,39]. This limitation is particularly relevant for acne therapy, where lesions are characterized by localized bacterial colonization and persistent perifollicular inflammation, and may therefore benefit from prolonged drug exposure at the diseased skin site rather than transient delivery alone [40]. In this context, a microneedle system capable of both efficient skin penetration and sustained intradermal release may offer distinct advantages.

Based on this rationale, we have designed and developed a sustained-release microneedle patch named Bacitracin@Hyaluronic Acid–Zein Microneedle (Bac@HA-ZMN). The antibacterial peptides loaded on the tips of Bac@HA-ZMN can effectively inhibit the activity of acne-related pathogenic bacteria. The tetrahedral needle tips with a length of 950 μm can withstand an axial pressure of 1.6 N, which is sufficient to penetrate the stratum corneum of the skin and continuously deliver the antibacterial peptides to the subcutaneous acne lesions through the microchannels locally formed by the needle tips. Bac@HA-ZMN significantly inhibited the proliferation of *P. acnes*, achieving a 57% reduction in acne area within 7 days and significantly reducing the area proportion of positive expression of inflammatory factors. Taken together, these results demonstrate that microneedles constructed from heterogeneous materials exhibit significant advantages over conventional homogeneous microneedles in terms of penetration depth and tissue-anchoring capacity, thereby conferring the structural foundation and functional benefits necessary for long-acting and precise transdermal drug delivery.

## 2. Materials and Methods

### 2.1. Materials

Hyaluronic acid (HA, MW 200–400 kDa), zein, glycerin, and bacitracin (Bac) were purchased from Shanghai Macklin Biochemical Technology Co., Ltd. (Shanghai, China). Ethanol was obtained from Sinopharm Chemical Reagent Co., Ltd. (Shanghai, China). *P. acnes* (ATCC 11827) was purchased from Shanghai Microbiological Culture Collection Co., Ltd. (Shanghai, China). The Assay Kit for Animal Live & Dead Cells was obtained from Thermo Fisher (China) Co., Ltd. (Shanghai, China). The Cell Counting Kit-8 was purchased from Beyotime Biotechnology Co., Ltd. (Shanghai, China). L929 cells were obtained from Haixing Biosciences (China). (Xiamen, China). The Bama miniature pig skin was purchased from Jiangsu Wanwei Biotechnology Co., Ltd. (Jiangsu, China).

### 2.2. Fabrication of HA-ZMN, Bac@HA-ZMN Microneedle Patches

#### 2.2.1. Fabrication of HA-ZMN

A total of 1.250 g of zein powder was weighed and dissolved in 5 mL of 80% ethanol solution. Then, 0.125 g of glycerol was added, and the mixture was thoroughly mixed using a magnetic stirrer at 1000 rpm. Subsequently, 20 μL of the mixed solution was drawn and evenly spread over the needle tips distribution area of the mold. The sample was vacuum-dried at 40 °C for 1 min to remove the solvent, and any residual liquid was wiped with clean, lint-free paper. This process was repeated until the needle tips were completely filled. Afterward, 1 mL of the base solution (3% HA) was added to the mold. The mold was placed in a vacuum drying oven at 40 °C until the material was fully dried and formed. Finally, the microneedle patch was removed from the mold for subsequent use.

#### 2.2.2. Fabrication of Bac@HA-ZMN

A total of 1.250 g of zein powder and 500 mg of Bac were weighed, and then 5 mL of an 80% ethanol aqueous solution was added. The mixture was stirred until fully dissolved, followed by the addition of 0.125 g of glycerol as a plasticizer. The mixture was then homogenized and prepared for use. The preparation of Bac@HA-ZMN was the same as that of HA-ZMN; however, the above mixture was used only to fill the needle tips, ensuring that only the needle tip part of the microneedle patch was loaded with Bac. After tip filling was completed, the residual solution outside the mold pinholes was removed, and the same base solution was injected. Then, 20 μL of the mixed solution containing bacitracin was drawn and evenly spread over the needle tips distribution area of the mold. The sample was vacuum-dried at 40 °C for 1 min to remove the solvent, and any residual liquid was wiped with clean, lint-free paper. This process was repeated until the needle tips were completely filled. Afterward, 1 mL of the base solution (3% HA) was added to the mold. The mold was then placed in a vacuum drying oven at 40 °C for about 16 h and left to stand until it was fully formed.

To ensure the reproducibility and batch-to-batch consistency of microneedle fabrication, all samples were prepared according to a standardized protocol under strictly controlled conditions. Key fabrication parameters, including drug concentration, casting volume, mold filling procedure, drying time, and drying temperature, were kept constant for all batches. In addition, the same microneedle molds and equipment settings were used during the entire preparation process. The fabricated microneedles were subsequently evaluated in terms of tip morphology, dimensional uniformity, and hydrophilicity to confirm the reproducibility of the preparation method and the consistency among different batches. The mold parameters are shown in Table 1.

#### 2.2.3. Calculation of Bac Loading in Bac@HA-ZMN

The loading amount of Bac in the Bac@HA-ZMN sample was determined and calculated by the density method. The solution from the needle tips during the preparation of the Bac@HA-ZMN sample was repeatedly filled and dried into a fixed volume centrifuge tube until the tube was full. Based on this, the density of the needle tips was calculated to be ρ = 1.147 g/cm^3^. Calculate the volume of a single needle tip using the parameters of the silicone mold, V = (S × h)/3 = 0.051 mm^3^ (needle tip height: 950 μm, side length: 390 μm). Therefore, the mass of 100 needle tips of a single sample is m = V × ρ × 100 = 5.8 mg. The Bac/zein mass ratio (2:5) was selected based on preliminary formulation optimization, considering both microneedle mechanical integrity and drug-release performance. Thus, the mass of Bac loaded on a single Bac@HA-ZMN sample is 1.66 mg.

### 2.3. Morphology Characterization of HA-ZMN Microneedle Patches

The microneedle samples were mounted on the sample stage and then placed in an ion sputter coater (MC1000, Hitachi High-Tech, Tokyo, Japan) to sputter-coat the surface with metal, enhancing conductivity. Subsequently, the surface morphology of the HA-ZMN microneedle patch was observed and analyzed using a scanning electron microscope (SEM, Regulus 8230, Hitachi High-Tech, Tokyo, Japan).

### 2.4. Compression Properties of HA-ZMN and Bac@HA-ZMN

The compression performance of the HA-ZMNs microneedle patch was tested using a tensile testing machine, and the displacement-force curve was plotted. During the test, the special-shaped material mode was selected under the compression module in the driver, with a gauge length of 1 mm (Universal Testing Machine (CMT-1104, SUST, Shenzhen, China)).

### 2.5. Swelling Properties of MN Patches

The ZMN microneedle patch was studied in terms of its swelling properties. The initial dry weight of the ZMN microneedle patch was recorded as m_0_. Subsequently, the ZMN patches were immersed in 15 mL of PBS (pH 7.4) at room temperature for 87 h. At predetermined time intervals, the patches were carefully removed, gently blotted dry with filter paper, and reweighed as m_t_. The swelling behavior of the ZMN microneedle patch was expressed as the percentage of swelling, according to the formula provided below. The detection period was defined according to the time point at which the mass of the patch approached a constant value. The calculation formula is detailed as follows:

(1) $$\%\mathrm{Swelling}\;=\;\frac{m_{t}-m_{0}}{m_{0}}\times 100\%$$

### 2.6. Hydrophilicity of MN Patches

The contact angle of the HA-ZMN microneedle patch was measured using a contact angle tester (DSA25E, Kruss, Hamburg, Germany). The intact HA-ZMN sample was placed steadily on the sample stage with the needle tips facing upwards. Then, the titrant was dispensed vertically onto the needle tips to accurately measure the contact angle. Meanwhile, the base part was cut from the HA-ZMN sample and placed steadily on the sample stage. Then, the same titration solution was dropped onto its surface to measure the contact angle. The volume of the titrant added in each case was 4 µL.

### 2.7. In Vitro Drug Release Study

During the experimental procedure, the transdermal diffusion apparatus (TP-6, Jintuo, Tianjin, China) was preheated to 37 °C to maintain a constant temperature; a magnetic stirrer was placed in the receiving chamber of the Franz diffusion cell, which was then completely filled with ultrapure water to eliminate any residual bubbles.

Excised Bama miniature pig skin was used to cover the receptor chamber (for microneedle samples, pre-compression was performed to ensure a secure fit and the control group consisted of a free bacitracin solution prepared from bacitracin powder (Shanghai Macklin Biochemical Co., Ltd., Shanghai, China), 1.66 mg/mL). The donor chamber of the Franz diffusion cell was placed on the skin surface and fixed with a metal clamp; 1 mL of Bac aqueous solution was added to the donor chamber of the control group. The assembled Franz diffusion cell was placed in the transdermal release instrument, and the magnetic stirring function was then activated. Aliquots (500 µL) of the sample were regularly collected through the sampling port and immediately replenished with the same volume of ultrapure water to maintain system balance. All experiments were conducted under the same conditions as those used for the microneedle group.

Upon completion of the experiment, the collected samples were analyzed using high-performance liquid chromatography (HPLC, 1260, Agilent Technologies, Santa Clara, CA, USA), and appropriate graphical visualizations were constructed to illustrate the results. The analytical conditions were configured as follows:

Chromatographic separation was performed using a ZORBAX SB-C18 column (4.6 mm × 250 mm, 5 μm; Agilent Technologies, Santa Clara, CA, USA) with a mobile phase composed of 0.1% formic acid in water and acetonitrile. The flow rate was maintained at 1 mL/min, the column temperature was set at 27 °C, the injection volume was 15 μL, and detection was carried out at a wavelength of 254 nm.

### 2.8. In Vitro Antibacterial Activity Assay

The liquid culture of *P. acnes* was conducted in Brain Heart Infusion (BHI) broth at 37 °C and 180 rpm in an orbital shaker for 48 h under aerobic conditions. For solid culture, BHI agar containing 1.5% (*w*/*v*) agar was used and incubated anaerobically at 37 °C for 48 h.

#### 2.8.1. Disk Diffusion Experiment

A total of 100 μL of *P. acnes* suspension (OD_600_ = 0.88) was added to the Petri dish, and the bacterial solution was evenly spread using glass beads. In the center of the Petri dish, place a drug sensitivity test paper (containing 20 µL of 1.66 mg/mL Bac solution), a blank microneedle patch (HA-ZMN), and a Bac-loaded microneedle patch (Bac@HA-ZMN). Another Petri dish inoculated with the same bacterial solution was used as the control group. All the Petri dishes were inverted and placed in an anaerobic incubator at 37 °C for 48 h, and then taken out and photographed as a record.

#### 2.8.2. Minimum Inhibitory Concentrations (MICs)

This study employed the microdilution method to determine the minimum concentration required to completely inhibit bacterial growth. In brief, 100 μL of Bac (1.66 mg/mL), HA-ZMN, and Bac@HA-ZMN (one microneedle patch/mL) were respectively added to each well of the first row of a 96-well plate, each of which contained 100 μL of sterile liquid medium. Subsequently, using the serial dilution method, a series of dilution solutions of different concentrations were prepared. Then, 10 μL of the diluted bacterial suspension was added to each well, and the mixture was incubated at 37 °C for 48 h with the aforementioned diluted compounds. The culture system without any compound was used as a negative control.

#### 2.8.3. Colony-Forming Unit (CFU) Reduction Assay

The antibacterial activity against *P. acnes* was evaluated by colony-forming unit (CFU) counting assays. For the CFU assay, bacterial suspensions after different treatments were cultured on agar plates, and the number of viable colonies was counted to calculate the CFU reduction rate relative to the blank control group.

### 2.9. In Vitro Cell Experiments

#### 2.9.1. In Vitro Cytotoxicity Test

L929 cells were cultured in a medium supplemented with 10% fetal bovine serum. And the experimental method was carried out in accordance with the operation guide of Cell Counting Kit-8. The culture bottles were taken out of the incubator. Under the microscope, it was confirmed that the cell morphology was spindle-shaped and they were adhering to the flask wall. The bottles were placed upright, and the culture medium was aspirated. 5 mL of PBS was added for rinsing 3–5 times. After removing the PBS, 1 mL of trypsin was added and the cells were digested horizontally for 1 min. Then, 2 mL of culture medium was added to the bottle, and the adherent cell layer was rinsed to ensure there was no residue. It was then transferred to a centrifuge tube and centrifuged at 1000 rpm/min for 1 min to remove the supernatant. A total of 3 mL of culture medium was added to the bottle, mixed, and 100 μL was taken and added to 900 μL of culture medium (diluted 10 times). Then, 10 μL was dropped onto the hemocytometer and the cell count was counted. Three 96-well plates were selected, with 6 replicate wells in each group. PBS solution was added to each well at the periphery of the plates. 100 μL of cell suspension (containing 3000–5000 cells/100 μL) was inoculated into each well. The culture plates were placed in a 37 °C, 5% CO_2_ incubator for 24 h. The original culture medium was discarded and replaced with fresh complete medium. The control group was added 10 μL of Bac solution (1.66 mg/mL) to each well, while the experimental groups were added 10 μL of HA-ZMN and Bac@HA-ZMN extract solution (concentration equivalent to 1 mg/mL). Cultures were maintained for 1, 2, and 3 days. At each time point, one 96-well plate was removed, the culture medium was aspirated, and 100 µL of 10% CCK-8 working solution was added to each well. The plates were incubated in the incubator for 1.5 h, and the absorbance values of each well were measured using an enzyme detector at a wavelength of 450 nm. The cell survival rate was calculated according to the following formula:

(2) $$\mathrm{Cell}\;\mathrm{survival}\;\mathrm{rate}\;=\;[(A_{t}-A_{0})/(A_{c1}-A_{0})]\times 100\%$$

A_t_: Experimental group;

A_0_: Blank group;

A_c1_: Co-incubation negative control group.

#### 2.9.2. In Vitro Cell Viability and Death Staining

The experimental method was carried out in accordance with the instructions of the Viability/Cytotoxicity Assay Kit for Animal Live & Dead Cells. Three single-cell culture dishes were prepared. A total of 1 mL of cell suspension (with a cell concentration of 30,000–50,000 cells/mL) was added to the experimental group and the control group respectively. Then, they were placed in the incubator for 24 h. The culture medium was discarded, and the dishes were washed twice with 1 mL of PBS buffer. After each addition, the PBS was gently aspirated. Subsequently, 100 μL of calcein AM and PI dyes (diluted 1:1000) were added, wrapped with tin foil to avoid light exposure, and incubated in the incubator for another 30 min. After the incubation, the culture dishes were taken out and imaged using a confocal laser scanning microscope (CLSM, TCS SP8, Leica Microsystems, Wetziar, Germany).

### 2.10. In Vivo Anti-Acne Performance of the MN Patches

The animal experimental process was approved by The Laboratory Animal Welfare and Ethics Committee of Zhejiang Huitong Testing and Evaluation Technology (Group) Co., Ltd. (Zhejiang, China). The animal experiment ethics approval number was HTDW-202502016, and approval was granted on 19 February 2025. All animals were maintained and used in accordance with the Animal Management Rules of the Ministry of Health of the People’s Republic of China and the Guide for the Care and Use of Laboratory Animals—Chinese Version. In addition, all animal experiments were conducted with the assistance of Zhejiang Huitong Evaluation Technology (Group) Co., Ltd. (Zhejiang, China).

#### 2.10.1. Establishment of *P. acnes*–Induced Mouse Acne Model

*P. acnes* were cultured in BHI liquid medium until the OD_600_ reached 0.85. After centrifugation at 6000 rpm for 10 min, the supernatant was discarded. Then, 10 mL of PBS buffer was added and centrifugation was performed at 6000 rpm for another 10 min. The supernatant was removed and this process was repeated twice to wash away any residual medium. Finally, 2.5 mL of PBS buffer was added to resuspend the bacteria into a suspension, which was set aside for later use. Male ICR mice were selected and their backs were shaved. Two injection sites were marked on either side of the spine, and 100 µL of the *P. acnes* suspension was injected into each site (20 mice in total). After 3 days, the growth of acne on the backs of the mice was observed.

#### 2.10.2. In Vivo Anti-Acne Performance Evaluation of Bac@HA-ZMN

A total of 20 model mice were selected and randomly divided into 4 groups, with 5 mice in each group (*n* = 5). The specific grouping and treatment were as follows: Group 1 was the control group and received no treatment; Group 2 placed Bac-sensitive tablets on the acne sites and fixed them on day 0; Group 3 was treated with HA-ZMN patches, which were pressed and fixed on the acne sites; and Group 4 was treated with Bac@HA-ZMN patches, which were also pressed and fixed on the acne sites. It should be noted that all experimental groups used 3M medical dressings for fixation. The specific operation was to press and attach the corresponding preparations to the acne sites of the mice and fix them with 3M tape (the day of treatment was designated as day 0). The acne conditions of the mice in each group were observed and analyzed on days 1, 3, and 7. In addition, several extra mice were prepared as backups in case of accidental death during the experiment; these animals were not included in the final statistical analysis.

During treatment with different methods, changes in the diameter of acne lesions were measured daily using a micrometer. On days 1, 3, and 7 of the experiment, as well as before euthanasia, changes in acne on the backs of the mice were photographed and recorded. Subsequently, skin samples were obtained by cutting 2 mm beyond the edge of the wound. The collected skin tissues were then successively fixed, dehydrated, cleared, paraffin-embedded, and sectioned for subsequent histological analysis.

Mice with successfully established acne-like lesions were randomly assigned to each treatment group. Because the dosage forms used in different groups were visually distinguishable, blinding was not applied during treatment administration. However, lesion observation and evaluation were performed according to the same criteria across all groups to reduce observational bias.

#### 2.10.3. Immunohistochemical Analysis of IL-8, MMP-2, and TNF-α

Skin tissues from the lesion sites were collected, fixed, paraffin-embedded, and sectioned for immunohistochemical analysis. The sections were deparaffinized in xylene, rehydrated through a graded ethanol series, and rinsed with distilled water. Antigen retrieval was performed using EDTA buffer (pH 9.0). Briefly, the sections were heated in the retrieval buffer until boiling, maintained for several minutes, and then allowed to cool to room temperature, followed by washing with PBST.

To block endogenous peroxidase activity, the sections were incubated with 3% H_2_O_2_ at room temperature for 25 min in the dark and then washed with PBST. After outlining the tissue area with a hydrophobic pen, the sections were blocked with normal serum at room temperature for 50 min. The sections were then incubated overnight at 4 °C with the following primary antibodies: anti-IL-8 (Abmart, Shanghai, China; 1:100), anti-MMP-2 (Proteintech, 1:100), and anti-TNF-α (Proteintech, Wuhan, China; 1:400). After washing with PBST, the sections were incubated with HRP-conjugated goat anti-rabbit secondary antibody (ZSGB-Bio, Beijing, China; 1:200) at 37 °C for 1 h. Immunoreactivity was visualized using DAB, and the nuclei were counterstained with hematoxylin. The sections were subsequently dehydrated, cleared, and mounted with neutral resin.

For quantitative analysis, the positive-stained area of IL-8, MMP-2, and TNF-α was measured using ImageJ (version 1.53a, National Institutes of Health, Bethesda, MD, USA) under identical microscope settings and threshold conditions. Five animals were included in each group (*n* = 5) for IHC analysis. The quantified data are expressed as mean ± SD and were statistically analyzed as described above.

#### 2.10.4. Lesion Area Quantification

The lesion area was quantified from digital photographs using ImageJ software under identical imaging conditions. The lesion boundaries were manually outlined, and the measured values were used for statistical analysis.

#### 2.10.5. Statistical Analysis

Data are presented as mean ± SD. Statistical analysis was performed using GraphPad Prism (version 10.6.0, GraphPad Software, San Diego, CA, USA). Differences among groups at each time point were analyzed using one-way ANOVA followed by Tukey’s multiple comparisons test. Only comparisons versus the control group are shown in the figure. A value of *p* < 0.05 was considered statistically significant.

## 3. Results

### 3.1. Fabrication and Characterization of the MN Patches

**Figure 1:**
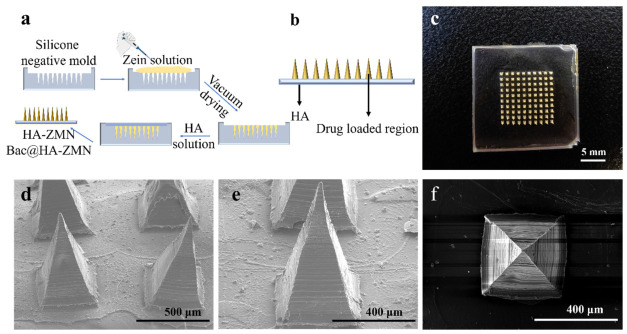
Preparation process and characterization of HA-ZMNs. (**a**) Schemic illustration of the preparation process of HA-ZMNs; (**b**) Diagram of Bac@HA-ZMN; (**c**) Photographs of HA-ZMNs; (**d**–**f**) SEM images of HA-ZMNs.

The microneedle patch tips were fabricated using a vacuum process, followed by casting the base solution. After drying and demolding, the complete samples were obtained (Figure 1). The sample was named HA-ZMN. If Bac was added to the zein solution, the resulting sample was named Bac@HA-ZMN. There was no obvious difference in appearance between the two samples.

The procedure for preparing the samples is depicted in Figure 1a, the functional segregation of the regions is shown in Figure 1b, and the appearance of the fabricated microneedle patch samples is illustrated in Figure 1c. SEM images (Figure 1d–f) further reveal that the tips of HA-ZMN were formed by the stacking of zein layers, presenting a regular tetrahedral shape with a tip height of 950 μm and a base diameter of 390 μm, and the HA base is smooth and uniform. The needle tips are of sufficient height to ensure penetration of the stratum corneum and successful delivery of the drug to the dermis. In addition, the truncated diameter of a single needle tip is less than 30 μm. This tiny and sharp geometric shape helps to effectively penetrate the skin. Compared with conventional microneedles, in which both the needle tips and base layer are composed of the same material, the present design employs heterogeneous materials for these two components, effectively enhancing the retention of the needle tips in the skin [41,42].

### 3.2. Physicochemical Properties of MN Patches

**Figure 2:**
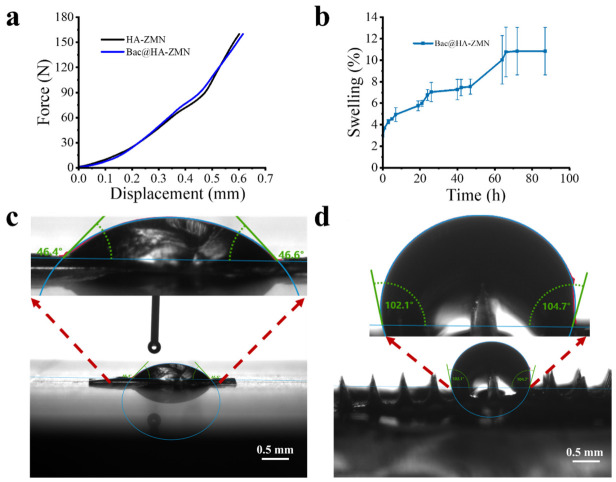
Characterization of the mechanical, swelling, and water contact angle of HA-ZMN and Bac@HA-ZMN. (**a**) Force-displacement curve of HA-ZMN and Bac@HA-ZMN; (**b**) Swelling percentage as a function of time of Bac@HA-ZMN; (**c**) Water contact angle of HA-ZMN substrate; (**d**) Water contact angle of HA-ZMN needle tips.

As shown in Figure 2a, the maximum force value of HA-ZMN is 160 N, and that of the Bac@HA-ZMN sample loaded with Bac is also 160 N. This result indicates that loading the drug has no significant impact on the mechanical strength of HA-ZMN. Additionally, the average maximum force of a single needle tip reached 1.6 N, which exceeds the minimum insertion force required to pierce skin (0.3 N per needle tip) [43,44]. Previous studies have shown that the force required for microneedle insertion into skin is generally in the sub-Newton range per needle and varies with microneedle geometry, tip characteristics, and skin conditions [45]. Moreover, Davis et al. demonstrated that the insertion force is substantially lower than the fracture force of microneedles [46,47]. Therefore, the measured mechanical strength in this study suggests that the fabricated microneedles have sufficient mechanical integrity for effective skin penetration. To further investigate the swelling performance of the HA-ZMN microneedle patch, its mass change before and after incubation in PBS was evaluated as a common method to assess the swelling capacity of materials. The swelling ratio of the microneedle patch was 10.8%, and it reached equilibrium after 60 h (Figure 2b). These findings suggest that, despite the overall hydrophobic nature of zein, its three-dimensional molecular network retains a certain degree of hydrophilic permeability, enabling interstitial fluid infiltration and thereby inducing slight swelling of the needle tips. This process may generate transient micropores or hydrated channels within the carrier, which provide a structural basis for the initial burst release of Bac.

To verify the differences in characteristics between the base and the tip of the microneedle patch, contact angle measurements were conducted on each respectively. As shown in Figure 2c,d, the contact angle of the base of the microneedle patch is 46.5° ± 0.1°, demonstrating strong hydrophilicity, while the contact angle at the tip of the needle is 103.4° ± 1.3°, indicating significant hydrophobicity, this finding further confirms the solubility of the base and the swelling property of the needle tip. The disintegration time of HA-ZMN in PBS solution also supports this observation (S1).

### 3.3. In Vitro Insertion and Drug Release of MN Patches

**Figure 3:**
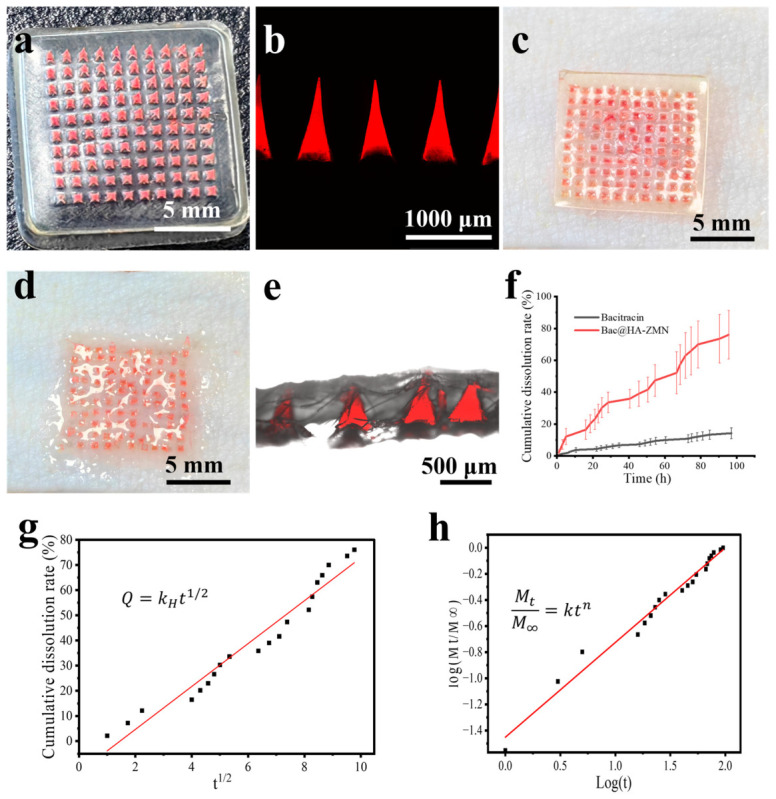
Transdermal evaluation of HA-ZMNs in the Bama miniature pig skin. (**a**) Photograph of HA-ZMNs stained with rhodamine; (**b**) CLSM image of HA-ZMN tips stained with rhodamine (emission: 570 nm, excitation: 550 nm); (**c**) Photograph of HA-ZMNs pressed on Bama miniature pig skin; (**d**) Photo of HA-ZMNs dissolved on the back of Bama miniature pig skin; (**e**) Microscope image showing depth of the needle tips penetrating Bama miniature pig skin; (**f**) Cumulative release of Bac from Bac@HA-ZMN as a function of time; Fitting of the in vitro release profile of Bac@HA-ZMN using the (**g**) Higuchi model and (**h**) Korsmeyer–Peppas model.

To track the penetration of Bac@HA-ZMN on the Bama miniature pig skin, a rhodamine-labeled microneedle patch was prepared (Figure 3a), and the integrity of the needle tips morphology was verified by CLSM (Figure 3b). Subsequently, the microneedle patch was pressed onto the surface of Bama miniature pig skin to evaluate its penetration performance (Figure 3c). The rhodamine-stained microneedle patches and the samples in Figure 3d were cut along the needle tips gaps and observed under a laser confocal microscope. The complete morphology of the needle tips and their insertion into Bama miniature pig skin could be clearly observed (Figure 3e). An insertion depth of approximately 500 μm is expected to reach the epidermal and superficial dermal follicular region, which is anatomically relevant to the pilosebaceous unit. Hair follicles are recognized as important penetration and reservoir pathways for locally delivered agents [48,49], and the pilosebaceous unit is a key therapeutic target in acne [50,51]. Therefore, this penetration depth supports the potential for localized delivery to the follicular/pilosebaceous microenvironment, although direct histological confirmation of sebaceous gland-level localization is still required. Although the microneedles had a geometric height of approximately 950 µm, the actual penetration depth in skin was only about 500 µm, corresponding to an insertion efficiency of ~52.6%. This difference is likely attributable to skin deformation, viscoelastic resistance, and incomplete insertion during application. Importantly, the actual insertion depth rather than the full needle height is more directly related to tissue stimulation and potential discomfort. Therefore, despite the relatively large needle height, the limited penetration depth observed here suggests a reduced likelihood of excessive deep dermal injury.

Meanwhile, after pressing the microneedle patch onto the Bama miniature pig skin and fixing it on the diffusion cell, the in vitro release of the drug it carried was measured. With Bac solution as the control, Figure 3f presents the in vitro release profile of Bac. Within 96 h, the cumulative release of free Bac was 14.2%, whereas the Bac@HA-ZMN microneedle patch achieved a higher cumulative release of 76.1%. To further elucidate the release mechanism of bacitracin from the drug-loaded microneedle tips, the in vitro release data were analyzed using the Higuchi and Korsmeyer–Peppas kinetic models. As shown in Figure 3g,h and summarized in Table S1 of supporting information, the release profile exhibited a good fit to the Higuchi model, with a Higuchi release constant (k_H_) of 8.5225 and a correlation coefficient of R^2^ = 0.9538. This result indicates that diffusion plays an important role in the release process of bacitracin from the microneedle matrix. Notably, the release data were better described by the Korsmeyer–Peppas model, which showed a higher correlation coefficient (R^2^ = 0.977) and a release exponent (n) of 0.72594. Since the value of n falls within the range of 0.45–0.89, the release behavior can be classified as anomalous (non-Fickian) transport, suggesting that the release process is not governed solely by Fickian diffusion, but rather by the combined effects of drug diffusion and polymer matrix relaxation/swelling. This interpretation is consistent with the physicochemical characteristics of zein, which serves as the drug-loaded matrix in the microneedle tips. Upon contact with the release medium, the zein matrix gradually absorbs water and undergoes swelling, resulting in partial loosening of the internal network structure. Such hydration-facilitated structural relaxation can promote the sustained diffusion of bacitracin from the microneedle tips into the surrounding medium. Therefore, the release of Bac from the zein-based microneedle tips is best described as a diffusion–swelling coupled sustained-release process.

In addition, the cumulative release of 76.1% within 96 h indicates that the microneedle system is capable of maintaining prolonged drug release over an extended period. This sustained-release behavior may be beneficial for localized anti-infective therapy by prolonging drug retention at the target site and reducing the need for frequent administration.

### 3.4. In Vitro Antibacterial Activity of MN Patches

**Figure 4:**
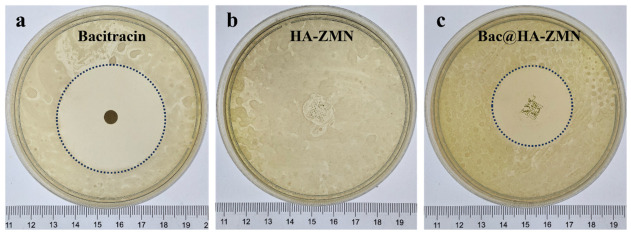
Antibacterial activity of HA-ZMNs against *P. acnes*. (**a**) Inhibition zone of Bac; (**b**) Inhibition zone of HA-ZMN; (**c**) Inhibition zone of Bac@HA-ZMN. Dashed circles mark the boundaries of the inhibition zones to facilitate area calculation. Brown dots indicate the needle tips of HA-ZMN and Bac@HA-ZMN.

The inhibition zone against *P. acnes* by Bac, HA-ZMN and Bac@HA-ZMN were measured by the disk diffusion assay (as shown in Figure 4). The antibacterial effects of Bac (Figure 4a), HA-ZMN (Figure 4b) and Bac@HA-ZMN (Figure 4c) were analyzed. The HA-ZMN without Bac showed only very small inhibition zones, whereas the inhibition zone of Bac@HA-ZMN was approximately 9.81 cm^2^, and that of the Bac control group was approximately 19.49 cm^2^. The inhibition zone area of Bac@HA-ZMN was approximately half that of the free Bac group, which is likely attributable to the limited adsorption capacity of the antimicrobial susceptibility discs and restricted diffusion in the agar medium, rather than a complete loss of antibacterial activity. These findings suggest that the zein microneedle tip, as a carrier, modulates Bac release without excessively hindering its availability.

The antibacterial activity against *P. acnes* was quantitatively assessed by both MIC (Table 2) and CFU assays (Table 3). Bac showed the lowest MIC (≤0.756 μg/mL), whereas HA-ZMN alone exhibited relatively weak antibacterial activity (48.4 μg/mL). After Bac loading, Bac@HA-ZMN showed an MIC of 12.1 μg/mL, indicating improved antibacterial activity. Consistently, the CFU assay showed that, compared with the blank control (3.2 × 10^9^ CFU), the Bac and Bac@HA-ZMN groups reduced bacterial counts to 1.97 × 10^9^ and 1.88 × 10^9^ CFU, corresponding to 38.44% and 41.56% reduction, respectively. Together with the previously obtained in vitro release profile, these MIC and CFU results suggest that bacitracin remains releasable and biologically active after incorporation into the microneedle system. The hydrophobic zein tips therefore appear to modulate the release kinetics rather than completely prevent drug release, supporting the sustained-release behavior of bacitracin in Bac@HA-ZMN.

### 3.5. In Vitro Cytotoxicity

**Figure 5:**
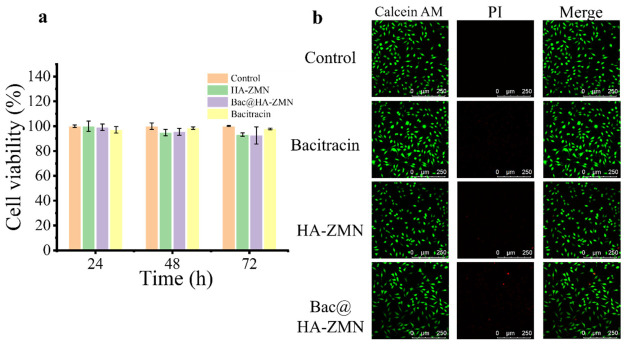
Cytocompatibility of the HA-ZMNs. (**a**) Cell viability of L929 cultured with various samples for 24 h, 48 h, 72 h; (**b**) CLSM images of L929 cultured with various samples.

Before further testing, we conducted an in vitro cytotoxicity assessment of Bac, HA-ZMN and Bac@HA-ZMN. The experiment utilized the L929 fibroblast cell line derived from mouse subcutaneous connective tissue, and cell viability was quantitatively analyzed using the CCK-8 kit. As shown in Figure 5a, the cells maintained good viability during the tests on days 1, 2, and 3. Specifically, the cells in the control group remained stably viable throughout the experiment; the cell viability in the blank HA-ZMN microneedle patch group decreased from 99.98% to 93.25% within 72 h; the cell viabilities in the Bac@HA-ZMN group on the three days were in the same trend as the HA-ZMN; the cell viability in the Bac group showed no significant changes within 72 h; while all above 90%. Additionally, Figure 5b shows that Bac, HA-ZMN, and Bac@HA-ZMN did not cause obvious damage to the cell morphology. Based on the above results, it can be concluded that the prepared microneedle patches have no significant cytotoxic on skin cells. The results of the live/dead cell staining assay further corroborated the aforementioned conclusion (Figure 5b).

### 3.6. In Vivo Evaluation of the Anti-Acne Effevts of MN Patches

**Figure 6:**
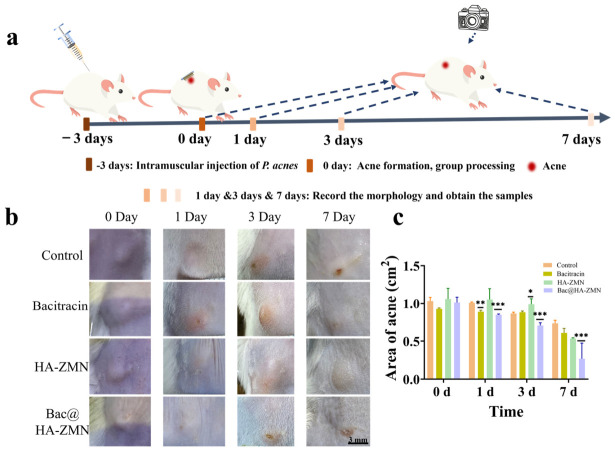
Alleviation of acne by different treatment. (**a**) Timeline for the establishment and evaluation of the acne mode; (**b**) Optical photographs of acne lesions showing morphological changes following different treatment; (**c**) Changes in the area of acne in each group. Data are presented as mean ± SD (*n* = 3). Statistical analysis at each time point was performed by one-way ANOVA followed by Tukey’s multiple comparisons test. * *p* < 0.05; ** *p* < 0.01; *** *p* < 0.001.

To evaluate the therapeutic effect of Bac@HA-ZMN patches on *P. acnes* infection, we established an acne model by subcutaneous injection *P. acnes* into the backs of ICR mice. Subsequently, all acne model mice were randomly divided into 4 groups (*n* = 5), and different treatment methods were applied to each group on day 0. The changes in acne were observed on days 1, 3, and 7 (Figure 6). According to different treatment intervention plans, the four groups of mice were respectively set as follows: the first group was the untreated control group, the second group received Bac treatment, the third group received blank microneedle patch HA-ZMN treatment, and the fourth group received the microneedle patch Bac@HA-ZMN treatment.

The raised acne appeared on the backs of the mice after subcutaneous injection of *P. acnes* for 3 days, indicating that the acne model was successfully established (Figure 6a). After the treatment on day 0, photographs of the lesion areas were taken on days 1, 3, and 7 to monitor changes in acne. In the untreated control group, the size of acne increased over time, and acne elevations were still visible after 7 days. In the Bac treated group, the same trend was observed, indicating that Bac could not effectively penetrate the skin barrier to inhibit the proliferation of *P. acnes* (Figure 6b). To further substantiate the gross observations, the acne lesion area was quantitatively measured at different time points after treatment. As shown in Figure 6c, the lesion area gradually decreased over time in all groups, with the most marked reduction observed in the Bac@HA-ZMN group. Statistical analysis revealed no significant difference among groups at day 0 (*p* = 0.2890), but significant differences were present at day 1 (*p* < 0.0001), day 3 (*p* = 0.0006), and day 7 (*p* = 0.0004). Compared with the control group, the Bac@HA-ZMN group showed significantly reduced lesion area at days 1, 3, and 7, while the Bac group showed a significant reduction only at day 1. These quantitative results were in agreement with the representative photographs, further supporting the superior therapeutic efficacy of Bac@HA-ZMN.

**Figure 7:**
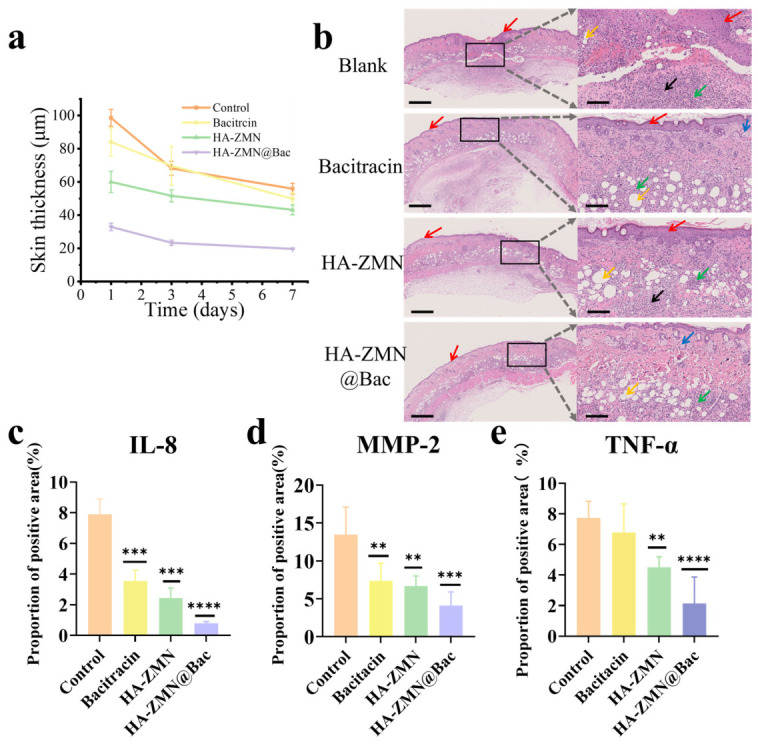
Histological and immunohistochemical analysis of acne lesions after different treatments. (**a**) Quantitative analysis of mice skin thickness change over time; (**b**) Representative microscopic images of hematoxylin and eosin (H&E) staining of the skin tissue sections from the mice after the different treatments (magnification, ×5 and ×20; scale bars, 500 and 100 μm, respectively). Epidermal thickening (red arrow), mild sebaceous gland hypertrophy in the dermis (blue arrow), severe inflammatory cell infiltration (green arrow), mild vacuolar changes (orange arrow), mild collagen fiber necrosis with fibrotic changes (black arrow) are indicated. (**c**–**e**) Proportion of positive area analysis of IL-8 protein (**c**), MMP-2 protein (**d**), and TNF-α protein (**e**) expression in the tissue from the acne area. Data are presented as mean ± SD (*n* = 3). Statistical analysis at each time point was performed by one-way ANOVA followed by Tukey’s multiple comparisons test. ** *p* < 0.01; *** *p* < 0.001; **** *p* < 0.0001.

The skin thickness at the acne sites decreased over time for every group, which was related to the self-healing function of the mice (natural recovery). Among them, the Bac@HA-ZMN group exhibited markedly stronger therapeutic effects than the other groups. These results confirm that Bac-loaded zein microneedles could effectively deliver the drug into the infected dermis, inhibit the proliferation of *P. acnes*, and accelerate the relief of acne symptoms following skin penetration (Figure 7a). On day 7, skin tissue from the acne lesions was collected, and hematoxylin and eosin (H&E) staining was performed for histological analysis. As shown in Figure 7b, the acne-affected regions of the control group displayed marked epidermal thickening. In the dermis, slight sebaceous gland enlargement was observed, accompanied by intense inflammatory cell infiltration, mild vacuolar degeneration, and large necrotic areas with granulation tissue hyperplasia; hair follicles were not observed. In the Bac group, the stratum corneum appeared thinned or even absent, while the epidermis exhibited marked thickening. Within the dermis, mild sebaceous gland enlargement was noted, along with intense inflammatory cell infiltration and moderate vacuolar degeneration. In the HA-ZMN group, epidermal thickening was evident, accompanied by mild sebaceous gland enlargement in the dermis, dense inflammatory cell infiltration, and slight vacuolar degeneration. Subcutaneous connective tissue hyperplasia, muscle atrophy, and mild collagen fiber necrosis with associated fibrosis were also observed. In the Bac@HA-ZMN group, no epidermal thickening was detected. Only mild inflammatory cell infiltration, subcutaneous connective tissue hyperplasia, slight vacuolar degeneration, and mild sebaceous gland hypertrophy were observed in the dermis. The Bac@HA-ZMN group exhibited the most favorable overall therapeutic efficacy, achieving a pronounced anti-inflammatory effect while effectively preserving the structural integrity of the skin tissue. By contrast, Bac treatment alone may impair the microbial barrier on the skin surface, thereby weakening the physical and immune barrier functions of the skin. Meanwhile, although the insertion of HA-ZMN microneedle tips allowed successful transdermal penetration, the lack of Bac delivery was insufficient to effectively inhibit the inflammatory response, which may in turn lead to fibrosis in the deeper dermis. In the acne model groups receiving different treatments, excessive proliferation of *P. acnes* is known to trigger and aggravate inflammation, stimulating keratinocytes to secrete IL-8, MMP-2, and TNF-α. The immunohistochemical findings are presented in Figure 7c–e, which clearly demonstrate the expression of these proteins in lesional skin. It is noteworthy that the Bac@HA-ZMN treatment group exhibited the lowest expression levels of inflammatory markers, with MMP-2 and TNF-α showing consistent down-regulation trends. These results suggest that Bac@HA-ZMN effectively facilitates Bac delivery and controlled release, thereby significantly suppressing the expression of pro-inflammatory cytokines and matrix-degrading enzymes, ultimately enhancing the overall therapeutic efficacy. Overall, the research findings reveal that Bac@HA-ZMN, through an efficient Bac delivery and controlled-release system, significantly suppresses the expression of pro-inflammatory cytokines and matrix-degrading enzymes, optimizes immune-cell infiltration patterns, and promotes orderly tissue repair. Consequently, it shifts the pathological progression of acne from destructive inflammation to controllable repair, highlighting its multi-target and multi-dimensional therapeutic advantages. This reduction in inflammatory marker expression should, however, be interpreted cautiously. Given the established antibacterial activity of bacitracin, the decreased levels of IL-8, MMP-2, and TNF-α are more likely attributable to the reduction in bacterial burden and the subsequent attenuation of infection-induced inflammation, rather than definitive evidence of a direct immunomodulatory effect. Since the present study was not specifically designed to distinguish antibacterial-mediated anti-inflammatory effects from direct immune regulation, the anti-inflammatory benefit observed here should be considered primarily secondary to bacterial clearance.

## 4. Conclusions

Acne is a chronic inflammatory skin disorder for which achieving sustained and stable therapeutic efficacy remains challenging. In the present study, we developed a detachable sustained-release microneedle system, Bac@HA-ZMN, based on a functionally compartmentalized design using heterogeneous materials. Specifically, the combination of a rapidly dissolvable HA base and mechanically robust hydrophobic zein tips enabled efficient skin penetration, stable in situ retention, and sustained local delivery of bacitracin. The resulting microchannels facilitated transdermal transport across the stratum corneum, while the zein matrix supported controlled drug release over an extended period. As a result, Bac@HA-ZMN effectively inhibited *P. acnes* proliferation, significantly reduced the expression of inflammatory cytokines, including IL-8, TNF-α, and MMP-2, and accelerated acne resolution in vivo. From a translational perspective, because topical antibacterial agents used in acne therapy are often limited by insufficient skin penetration, repeated application, and local irritation, Bac@HA-ZMN may offer a promising localized therapeutic strategy by physically bypassing the skin barrier, improving local bacitracin delivery to acne-associated tissue, and enabling long-acting antibacterial treatment. In addition, compared with systemic antibiotic therapy, this localized microneedle-mediated delivery approach may help reduce unnecessary systemic exposure. However, conventional topical therapies remain noninvasive and convenient for routine use, and the present study did not include a direct comparison with conventional topical bacitracin formulations, such as creams or ointments. Therefore, the practical advantages of Bac@HA-ZMN, including its potential benefits in local drug deposition, sustained release, patient compliance, and clinical acceptability, still require further validation in future head-to-head comparative studies. Another important consideration for the potential clinical translation of Bac@HA-ZMN is the risk of antibiotic resistance. As bacitracin is an antibiotic, repeated or prolonged exposure may still impose selective pressure on bacteria. Although localized delivery may improve drug deposition at the lesion site and potentially reduce the overall antibiotic burden, it cannot completely eliminate the possibility of resistance development. Therefore, future studies should further investigate the long-term resistance risk of Bac@HA-ZMN through serial passage, repeated-exposure, and susceptibility evaluation against *P. acnes*. Overall, this work demonstrates a promising microneedle-based platform for localized acne treatment and may provide a useful strategy for the sustained transdermal delivery of antimicrobial peptides in cutaneous diseases.

## Figures and Tables

**Table 1:** Specifications of the mold used for the fabrication of Bac@HA-ZMN microneedle patches.

Needle Tip Shape	Needle Tip Height (μm)	Base Side Length (μm)	Array Arrangement
Quadrangular pyramid	950	390	10 × 10

**Table 2:** MICs of Bac, HA-ZMN and Bac@HA-ZMN.

Compound	MIC (μg/mL) *
	*P. acnes* (ATCC 11827)
Bacitracin	≤0.756
HA-ZMN	48.4
Bac@HA-ZMN	12.1

* MICs were the mean value of three independent experiments.

**Table 3:** Colony-forming unit (CFU) reduction assay of different treatments against *P. acnes*.

Treatment Group	Colony-Forming Units (CFU)	CFU Reduction Rate (%)
Control	3.2 × 10^9^	—
Bac	1.97 × 10^9^	38.44
Bac@HA-ZMN	1.88 × 10^9^	41.56

## Data Availability

The original contributions presented in this study are included in the article/Supplementary Material. Further inquiries can be directed to the corresponding authors.

## Supplementary Materials

The following supporting information can be downloaded at: https://www.mdpi.com/article/10.3390/polym18101250/s1, Figure S1: Dispersion behavior of HA-ZMNs in water. (**a**) Initial morphology of HA-ZMNs; (**b**) HA-ZMNs immediately after immersion in water (0 s); (**c**) HA-ZMNs after 30 s of immersion in water; (**d**) HA-ZMNs after 60 s of immersion in water; Figure S2: SEM images of Bac@HA-ZMN from different batches; Figure S3: Water contact angle of Bac@HA-ZMN needle tips; Table S1: Kinetic fitting parameters of bacitracin release from Bac@HA-ZMN.
