# Supplementary material for: Sustained-Release Microneedles for Local Delivery of Antibacterial Peptide in Acne Therapy
Source: Polymers (Basel). 2026 May 21;18(10):1250. doi: 10.3390/polym18101250 (PMC13211109; doi:10.3390/polym18101250)
Supplement: Supplementary file 1 [file polymers-18-01250-s001.zip › polymers-4273836-supplementary.pdf]

Supporting Information

Sustained-Release Microneedles for Local Delivery of Antibacterial Peptide in Acne Therapy

Jingyu Gao[1,2,3], Zhangyong Si[1,2], Mengdi Xu[1,2], Shengyu Zhang[1,2], Fan Fan[1,2], Feng Zhou[1,2*], Jiantao Zhang[2,3*]

1: Laboratory of Advanced Theranostic Materials and Technology, Ningbo Institute of Materials Technology and Engineering, Chinese Academy of Sciences, Ningbo 315300, China

2: Ningbo Cixi institute of Biomedical Engineering, Ningbo 315300, China

3: Zhejiang Key Laboratory of Biopharmaceutical Contact Materials, Ningbo 315300, China

* Correspondence: zhoufeng@nimte.ac.cn; zhangjiantao@nimte.ac.cn

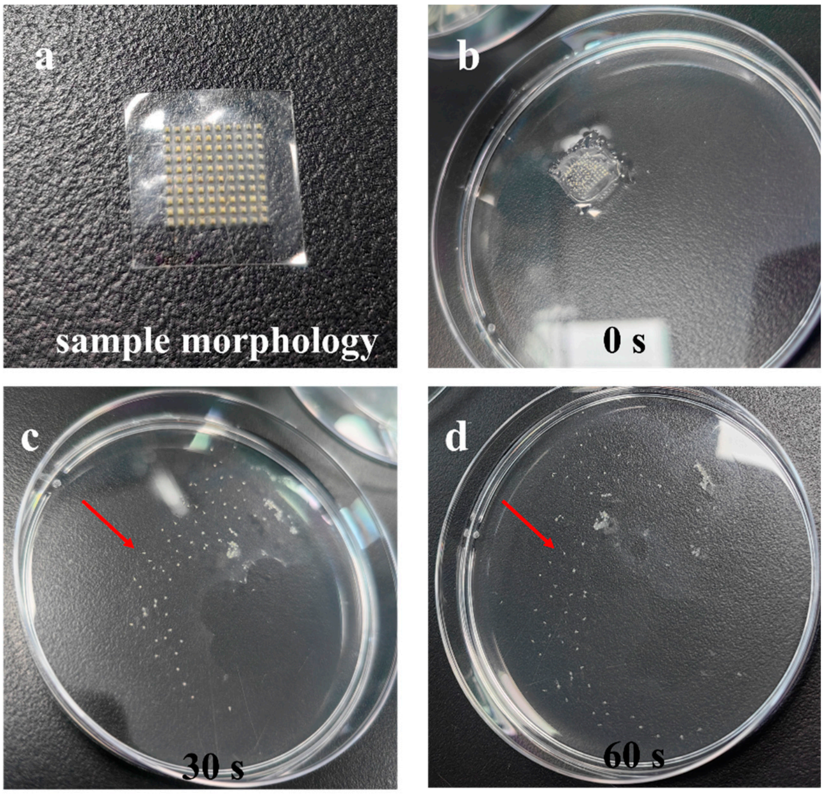

Figure S1. Dispersion behavior of HA-ZMNs in water. (a) Initial morphology of HA-ZMNs; (b) HA-ZMNs immediately after immersion in water (0 s); (c) HA-ZMNs after 30 s of immersion in water; (d) HA-ZMNs after 60 s of immersion in water.

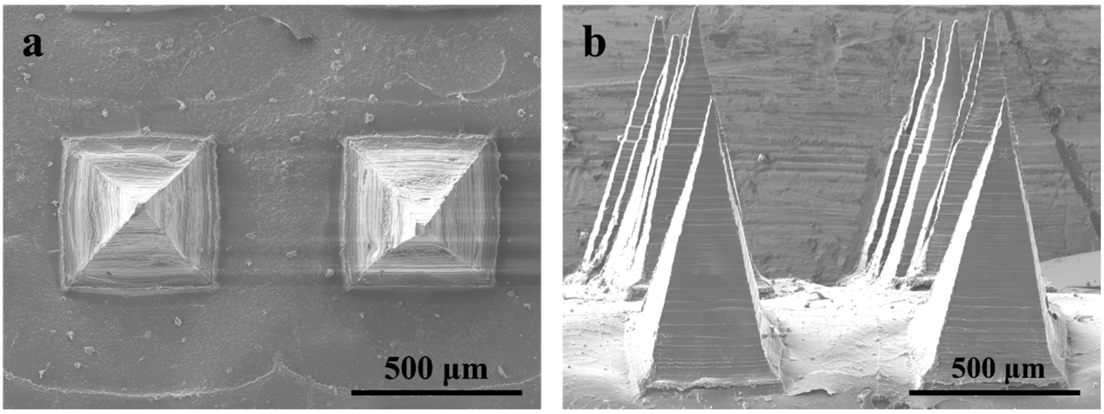

Figure S2. SEM images of Bac@HA-ZMN from different batches.

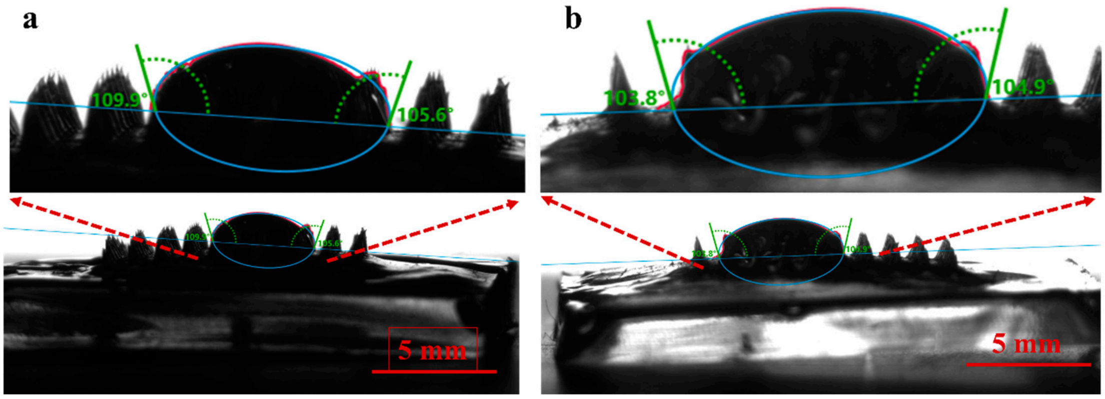

Figure S3. Water contact angle of Bac@HA-ZMN needle tips.

Table S1. Kinetic fitting parameters of bacitracin release from Bac@HA-ZMN.

Model	Equation	Parameter(s)	R^2
Higuchi	$Qt = k_H t^{1/2}$	k_H =8.5225	0.9538
Korsmeyer–Peppas	$M_t/M_\infty = kt^n$	n =0.72594	0.977
